# Synthesis Methods and Optical Sensing Applications of Plasmonic Metal Nanoparticles Made from Rhodium, Platinum, Gold, or Silver

**DOI:** 10.3390/ma16093342

**Published:** 2023-04-24

**Authors:** Elizaveta Demishkevich, Andrey Zyubin, Alexey Seteikin, Ilia Samusev, Inkyu Park, Chang Kwon Hwangbo, Eun Ha Choi, Geon Joon Lee

**Affiliations:** 1 Research and Educational Center, Fundamental and Applied Photonics, Nanophotonics, Immanuel Kant Baltic Federal University, 236016 Kaliningrad, Russia; 2 Department of Physics, Amur State University, 675021 Blagoveshchensk, Russia; 3 Department of Physics, University of Seoul, Seoul 02504, Republic of Korea; 4 Department of Physics, Inha University, Incheon 22212, Republic of Korea; 5 Department of Electrical and Biological Physics, Kwangwoon University, Seoul 01897, Republic of Korea; 6 Plasma Bioscience Research Center, Kwangwoon University, Seoul 01897, Republic of Korea

**Keywords:** surface plasmon, surface-enhanced Raman scattering, metal-enhanced fluorescence, ultraviolet plasmonics, platinum nanoparticles, rhodium nanoparticles, gold nanoparticles, silver nanoparticles, optical sensing

## Abstract

The purpose of this paper is to provide an in-depth review of plasmonic metal nanoparticles made from rhodium, platinum, gold, or silver. We describe fundamental concepts, synthesis methods, and optical sensing applications of these nanoparticles. Plasmonic metal nanoparticles have received a lot of interest due to various applications, such as optical sensors, single-molecule detection, single-cell detection, pathogen detection, environmental contaminant monitoring, cancer diagnostics, biomedicine, and food and health safety monitoring. They provide a promising platform for highly sensitive detection of various analytes. Due to strongly localized optical fields in the hot-spot region near metal nanoparticles, they have the potential for plasmon-enhanced optical sensing applications, including metal-enhanced fluorescence (MEF), surface-enhanced Raman scattering (SERS), and biomedical imaging. We explain the plasmonic enhancement through electromagnetic theory and confirm it with finite-difference time-domain numerical simulations. Moreover, we examine how the localized surface plasmon resonance effects of gold and silver nanoparticles have been utilized for the detection and biosensing of various analytes. Specifically, we discuss the syntheses and applications of rhodium and platinum nanoparticles for the UV plasmonics such as UV-MEF and UV-SERS. Finally, we provide an overview of chemical, physical, and green methods for synthesizing these nanoparticles. We hope that this paper will promote further interest in the optical sensing applications of plasmonic metal nanoparticles in the UV and visible ranges.

## 1. Introduction

**Figure 1:**
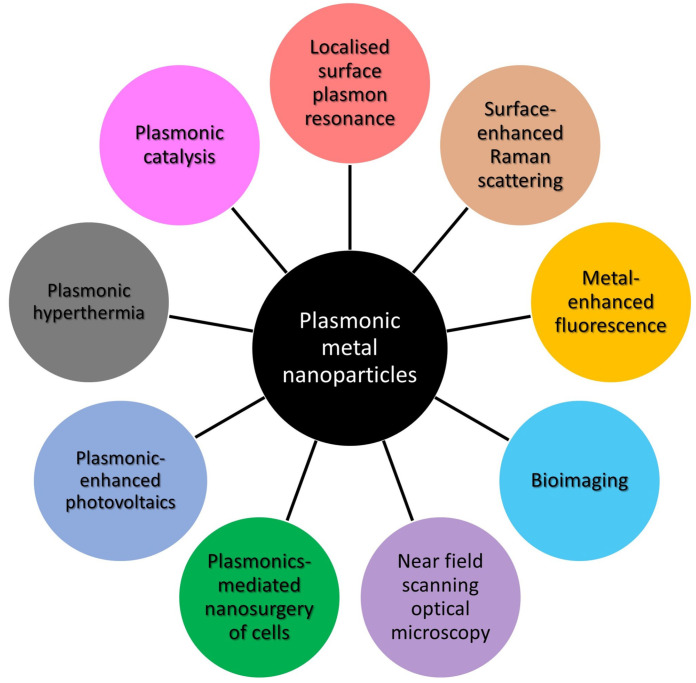
Potential applications of plasmonic metal nanoparticles.

Plasmonics deals with the free electron vibrations in metal nanostructures, and the interaction of such vibrations with atoms and molecules to create optical nanodevices. A surface plasmon is a collective oscillation of free electrons on the metal surface when the real part of the metal dielectric constant is negative. If the electromagnetic wave is coupled to collective electron oscillations, a surface plasmon wave is induced and propagates along the metal surface, and the electric field in the normal direction to the metal surface is nonradiative and strongly localized at the metal surface [1,2,3,4]. An important phenomenon in plasmonics is the strong spatial localization of electron oscillations at a plasmon resonance frequency. This strong localization leads to a huge increase in the local electric and magnetic fields. Localized surface plasmon resonance (LSPR) is the collective oscillation of free electrons like that of surface plasmon resonance (SPR) [3,4]. LSPR comes into play when light interacts with metal nanoparticles (NPs) with sizes less than the wavelength of incident light, and the oscillation is a standing wave of electron density confined near the particle [2,3,4,5,6]. Plasmonic metal nanoparticles have received great interest due to various applications, such as optical sensors, surface-enhanced Raman scattering, metal-enhanced fluorescence, metasurface devices, plasmonic catalysis, plasmonic hyperthermia, plasmonic-enhanced photovoltaics, plasmonic nanoantennas, near-field scanning optical microscopy, subwavelength plasmonic optics, theranostics, and biomedical imaging [7,8,9,10,11] (Figure 1).

The properties of localized surface plasmons strongly depend on the shape, size, and composition of metal nanoparticles [12,13], and these make it possible to tune their resonance frequencies for effective interactions of light with elementary quantum systems, such as molecules and quantum dots. In past years, tuning LSPR of metal nanoparticles by changing their shape and composition has been one of the most reliable and promising ways to obtain the necessary plasmon characteristics for sensor applications. Enhanced local fields near nanoparticles lead to an increase in the Raman scattering intensity by an order of $10^{14}$, making it possible to detect individual molecules [14,15]. The plasmon-enhanced local fields can lead to the development of methods for determining the structure of deoxyribo-nucleic-acids (DNA) without attaching markers to them [16]. Using plasmonic nanoparticles with complex structures, it is possible to simultaneously enhance both the absorption and emission of light and thus create effective fluorophores and nanosized light sources [17]. Metal nanostructures have been employed in plasmon-enhanced optical sensing applications such as surface-enhanced Raman scattering (SERS), metal-enhanced fluorescence (MEF), and biomedical imaging [3,5,18,19,20] (Figure 1).

In this paper, we review fundamental concepts, optical sensing applications, and synthesis methods of plasmonic metal nanoparticles made from rhodium (Rh), platinum (Pt), gold (Au), or silver (Ag). In Section 2 and Section 3, fundamental concepts and optical sensing applications of metal nanoparticles are demonstrated. To understand how metal nanoparticles are used for optical sensing, we explain plasmonic enhancement of the optical field near metal nanoparticles by electromagnetic theory. For various nanoscale materials, plasmonic enhancement near metal nanomaterials is also described by the finite-difference time-domain (FDTD) numerical simulation method. Then, we provide a review of the optical sensing applications of plasmonic metal nanoparticles. Biophysical and ultraviolet (UV) plasmonic applications of metal nanoparticles are described for Rh and Pt nanoparticles. Rh- and Pt-nanoparticle-based colloidal and planar optical sensors are described for MEF and SERS applications. Development of sensing methodologies and practical applications are reviewed for Rh and Pt nanoparticles. Optical sensing applications of metal nanoparticles in materials and biomedicine are described for Au and Ag nanoparticles. Gold nanoparticle-based optical sensing is described for the SERS detection of cancer cells. Polarization-dependent SERS and gap-enhanced Raman scattering characteristics are demonstrated to achieve the highly selective and sensitive nanoprobe. We also review practical applications of plasmonic metal nanoparticle-based optical sensing such as the discrimination of food-borne pathogenic microorganisms and environmental contaminant monitoring. Major synthesis methods of these metal nanoparticles are reviewed in Section 4 and Section 5.

## 2. Theoretical Background for Plasmon-Enhanced Optical Sensing of Metal Nanoparticles

When a light wave interacts with metal nanoparticles, the optical field is modified by the plasmon resonance effects of nanoparticles. Plasmonic enhancement near metal nanomaterials is explained by electromagnetic theory. In the long wavelength limit where the wavelength (λ) of the incident light is much larger than the particle diameter (*R*), the local electric field near the particle, $E_{loc}$, is given by

(1) $$\begin{array}{ccc} & & {\vec{E}}_{loc}\left(x,y,z\right)\;=\;E_{0}\;\hat{z}-\alpha \;E_{0}\left[\frac{\hat{z}}{r^{3}}-\frac{3z}{r^{5}}\;\vec{r}\right], \end{array}$$

where *r* is the radial distance from the particle center, $\vec{r}=x\hat{x}\;+\;y\hat{y}\;+\;z\hat{z}$, and α is the polarizability. We assumed that the incident light is *z*-polarized (${\vec{E}}_{inc}=E_{0}\;\hat{z}$). Here, $E_{0}$ is the magnitude of incident electric field. The first term in Equation (1) is the applied field, and the second is the induced dipole field. The polarizability describes the distortion of the electron cloud of a molecule by an external electric field and is defined by [21,22,23]:

(2) $$\begin{array}{ccc} & & \alpha \;=\;g\;4\pi \varepsilon_{d}R^{3}, \end{array}$$

where *g* is defined as

(3) $$\begin{array}{ccc} & & g\;=\;\frac{\varepsilon_{m}-\varepsilon_{d}}{\varepsilon_{m}+2\varepsilon_{d}}\;=\;\frac{\left(\varepsilon_{m(Re)}-\varepsilon_{d}\right)\;+\;i\;\varepsilon_{m(Im)}}{\left(\varepsilon_{m(Re)}+2\varepsilon_{d}\right)\;+\;i\;\varepsilon_{m(Im)}}. \end{array}$$

Here, $\varepsilon_{m}$ and $\varepsilon_{d}$ are the dielectric constants of the metal nanoparticle and the surrounding dielectric medium, respectively. $\varepsilon_{m(Re)}$ and $\varepsilon_{m(Im)}$ are the real and imaginary parts of the complex dielectric constant ($\varepsilon_{m}$) of the metal nanoparticle, respectively ($\varepsilon_{m}=\varepsilon_{m(Re)}+i\varepsilon_{m(Im)}$). The maximum magnitude of polarizability is obtained when the real part of the denominator in Equation (3) goes to zero, corresponding to a metal dielectric constant whose real part is $-2\varepsilon_{d}$.

According to Mie scattering theory, the scattering ($\sigma_{sca}$) and absorption ($\sigma_{abs}$) cross-sections of spherical metal nanoparticles are defined as [18,21,23]:

(4) $$\begin{array}{ccc} & & \sigma_{sca}\;=\;{\frac{8\pi }{3}k^{4}R^{6}\left|\frac{\varepsilon_{m}-\varepsilon_{d}}{\varepsilon_{m}+2\varepsilon_{d}}\right|}^{2}, \end{array}$$

(5) $$\begin{array}{ccc} & & \sigma_{abs}\;=\;4\pi kR^{3}\;Im\left(\frac{\varepsilon_{m}-\varepsilon_{d}}{\varepsilon_{m}+2\varepsilon_{d}}\right), \end{array}$$

where k=2π/λ. The plasmon resonance effects occur for scattering and absorption when the Fröhlich condition ($\varepsilon_{m(Re)}=-2\varepsilon_{d}$) is satisfied [21]. These resonances are due to resonant excitation of the dipole surface plasmon.

Fluorescence or Raman scattering intensities can be enhanced dramatically when some molecules exist near metal nanoparticles. These phenomena lead to SERS and MEF. The SERS enhancement factor (*G*) is given by [18,24,25]

(6) $$\begin{array}{ccc} & & G\;=\;|E_{loc}\left(\omega_{Ex},r\right){|}^{2}\;{\left|E_{loc}\left(\omega_{R},r\right)\right|}^{2}, \end{array}$$

where $\omega_{Ex}$ and $\omega_{R}$ are the frequency of the excitation and Raman-scattered light, respectively. $E_{loc}\left(\omega_{Ex},r\right)$ is the local electric field of excitation light near the metal nanoparticles, and $E_{loc}\left(\omega_{R},r\right)$ is the local electric field of Raman-scattered light near the nanoparticles. Assuming a small frequency shift between the excitation and Raman-scattered light ($\omega_{Ex}\approx \omega_{R}$), the Raman enhancement performance is approximately proportional to the fourth power of the local electric field, the so-called ${\left|E\right|}^{4}$ approximation [18,24],

(7) $$\begin{array}{ccc} & & G\;=\;|E_{loc}\left(\omega_{R},r\right){|}^{4}. \end{array}$$

MEF occurs when fluorophores are located at nanometric distances (up to 100 nm) from metallic surfaces. MEF is a complex coupling process between fluorophores and metal nanostructures, which leads to the modification of near- and far-field optical effects [26]. MEF is also referred to as surface-enhanced fluorescence (SEF). Assuming that there is no saturation effect in the excited state, the fluorescence emission power ($P_{em}$) is proportional to the excitation photon flux ($F_{ex}$) [27]

(8) $$\begin{array}{ccc} & & P_{em}\;=\;Q\;\sigma_{abs}\;F_{ex}, \end{array}$$

where *Q* is the quantum yield of the fluorophore, and $\sigma_{abs}$ is the absorption cross-section of the fluorophore. In the presence of metal nanoparticles, the local excitation light flux ($F_{loc}$) is given by $F_{loc}=\left(E_{loc}/E_{inc}\right)\;F_{ex}$, where $E_{loc}$ is the local electric field near metal nanoparticle [27]. In addition, fluorescence emission can be enhanced by modification of the radiative decay rate of the fluorophore through the local modification of the photon density of states; with an increased radiative rate, the lifetime of the fluorophore decreased [27]. The fluorescence intensity can be increased up to 100 times due to the plasmon-enhanced local field. Meanwhile, fluorescence emission can be quenched for fluorophores at short distance (<5 nm) from the metal surface or in direct contact with the metal surface, in which the quenching effect overwhelms the enhancement effect. Different mechanisms for the fluorescence enhancement and quenching of metal nanoparticles have been suggested, but the precise mechanism is still unknown due to the complexity of metal–fluorophore interactions [26,27]. The basics of MEF and it applications in biophysics can be found in the reference articles by Lakowicz et al. [28] and Aslan et al. [29,30].

For various nanoscale materials such as nanospheres, nanoshells, nanoellipsoids, nanocubes, and many others, there are several numerical tools available to calculate and simulate the spatial distribution of the local electric field intensity near the metal nanoparticles. The most common method is the FDTD method [31]. Solving Maxwell’s equations using finite-difference approximation in time and space (as in FDTD) is a widely used technique in studying electromagnetic waves and light traveling in a medium. FDTD is particularly useful for studying the optical properties of various nanoparticles [32,33,34,35], and has been used to investigate near-field optical properties of nanoscale materials [36], near-field probes [37], reflectometry-based plasmonic biosensors [38], nanoparticle structures for solar cell efficiency enhancement [39,40] and for SERS [41]. Another useful and well-used numerical method is the finite element method (FEM), which solves Maxwell’s equations by discretizing the geometry. FEM is generally known to be more efficient for electromagnetic problems with complex nanostructures [42,43]. It has been used to study far- and near-field optical properties of metal nanostructures with complex geometry [44,45], as well as to design nanoparticle structures for metal-enhanced fluorescence [46,47] and for SERS [48], metal–insulator–metal (MIM) waveguide for refractive index sensing applications [49], and bio-related applications, e.g., hyperthermia [50], photothermal therapy [51,52], and hydrolysis [53]. There is also the discrete dipole approximation (DDA) method. In this method, metal nanoparticles are treated as collections of tiny dipoles, and their optical scattering and absorption properties are calculated [54]. A quantum mechanical approach, such as density functional theory (DFT), can also be employed to investigate the optical, optoelectronic, catalytic, and magnetic properties of nanoparticles [55]. This method is particularly useful for studying the electronic structure of metal nanoparticles, which are difficult to model using classical approaches [55]. Each of these methods has its own strengths and limitations. Therefore, it is important to select an appropriate method considering the nature of the problem to be solved and the computational accuracy to be achieved.

In this review, we will briefly introduce some results from FDTD calculations for the optical properties of nanoparticles. The Maxwell equations are given by [56]

(9) $$\begin{array}{ccc} & & \nabla \times \vec{E}\;=\;-\mu_{0}\;\mu_{r}\;\frac{\partial }{\partial t}\vec{H} \end{array}$$

(10) $$\begin{array}{ccc} & & \nabla \times \vec{H}\;=\;+\varepsilon_{0}\;\varepsilon_{r}\;\frac{\partial }{\partial t}\vec{E}\;+\;\sigma \vec{E}. \end{array}$$

Here, $\vec{E}$ and $\vec{H}$ are the electric and magnetic fields, respectively. $\varepsilon_{0}$ and $\mu_{0}$ are the vacuum permittivity and the vacuum permeability, respectively. $\varepsilon_{r}=\varepsilon /\varepsilon_{0}$ is the ratio of the permittivity of a material to the vacuum permittivity. $\mu_{r}=\mu /\mu_{0}$ is the ratio of the permeability of a material to the vacuum permeability, and σ is the electric conductivity. In FDTD, the electromagnetic fields are discretized in both space and time, and the Maxwell equations are solved iteratively in a finite region of space. In the method, the spatial derivatives of the electric fields are approximated as the differences between neighboring grid points.

$$\frac{\partial E_{x}}{\partial x}\approx \frac{E_{x(i,j,k)}^{n}-E_{x(i-1,j,k)}^{n}}{\Delta x}$$

where $E_{x(i,j,k)}^{n}$ represents the value of the *x*-component of the electric field at the grid point (i,j,k) and time step (n). Δx is the grid spacing along the *x*-axis. As for the time derivative of the magnetic field, we can write

$$\frac{\partial H_{x}}{\partial t}\approx \frac{H_{x(i,j,k)}^{n+1}-H_{x(i,j,k)}^{n}}{\Delta t}$$

where $H_{x(i,j,k)}^{n}$ represents the value of the magnetic field at the grid point (i,j,k) and time step (n). In this way, in the FDTD method, the time displacement of the magnetic field makes the change of the electric field, and the finite change of the electric field makes the displacement of the magnetic field, and Maxwell’s equation is solved by tracing these changes in time.

**Figure 2:**
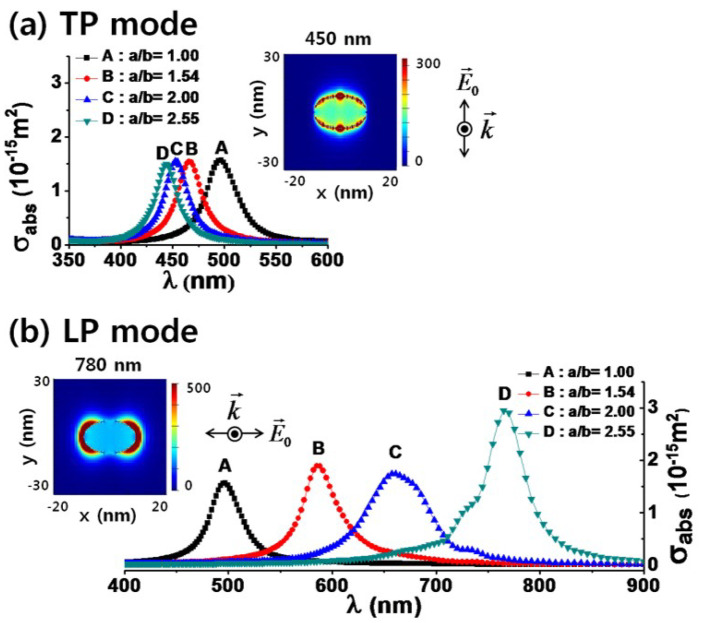
Absorption spectra of transverse plasmon (TP) and longitudinal plasmon (LP) for silver nanoellipsoids with four different aspect ratios. The inset figures show the spatial distributions of optical intensity for the nanoellipsoid with an aspect ratio of 2.55. Reprinted with permission from ref. [34]. Kim, J. et al., *Journal of Nanoscience and Nanotechnology* **12**, 5527 (2012). © 2012 American Scientific Publishers.

Figure 2 shows the spatial intensity distribution and absorption spectra near the Ag NP [34]. The spatial intensity distribution and absorption spectra were obtained by solving Maxwell equations using the FDTD method [31,34,35]. The aspect ratios of nanoellipsoids in A, B, C, and D were 1.00, 1.54, 2.00, and 2.25, respectively. Here, the aspect ratio represents the ratio of major axis to minor axis of the ellipsoid. The volumes of four different nanoellipsoids were equivalent to a nanosphere with a diameter of 15 nm. We can observe local enhancement of the optical field near the surfaces of Ag NPs. Transverse plasmon (TP) and longitudinal plasmon (LP) peaks appeared as the shape of nanoparticle varied from a sphere to an ellipsoid, and their spectral spacing will increase as the aspect ratio increases. Therefore, ellipsoidal metal nanoparticles exhibit strong polarization-dependent absorption spectra, where a small change in the aspect ratio led to a significant shift of the plasmon resonance band. For spherical nanoparticles, the SPR wavelength of nanoparticles was red-shifted with increasing nanoparticle size [34].

## 3. Optical Sensing Applications of Rhodium, Platinum, Gold, and Silver Nanoparticles

### 3.1. Rhodium- and Platinum-Nanoparticle-Based Optical Sensing

Traditionally, noble metals such as Au, Ag, and Cu are employed for the synthesis of nanoparticles [30,57,58,59,60,61,62,63,64]. The applications of these nanoparticles are limited to the visible and near-infrared (NIR) wavelength ranges. A variety of biological substances have fluorescence emission in the UV region, which require UV–MEF measurements. Furthermore, there is no fluorescence emission in the deep UV region, which makes it possible to effectively apply the UV–SERS technique for biomedical sensing. Recent studies have shown that Al, Ga, Mg, and Rh are promising materials for UV plasmonics [65,66]. At present, aluminum, being less expensive, is commonly used for studies in the UV and deep UV regions [67]. However, it has an oxide film and is sensitive to ambient temperature and humidity. Therefore, the plasmon-resonance-peak wavelengths for aluminum nanoparticles can change spectrally. Using experimental and theoretical approaches, authors showed that the formation of an aluminum oxide layer led to both a red-shift and a weakening of resonance peaks for aluminum nanoparticles of various shapes [68,69]. Magnesium also has an absorption maximum in the UV region, but it oxidizes much more than aluminum, so it is more difficult to realize UV plasmonic applications of magnesium nanoparticles. Some metals are resistant to oxidation. For example, gallium does not oxidize, is stable over a wide temperature range, and retains its stability for several years; therefore, it can be used for such studies [70]. Platinum and rhodium are among the most interesting metals to work with in UV plasmonics. These metals have a strong plasmon response in the UV region and can be used for UV plasmonic applications [71]. These metals do not oxidize, and they have practically no oxide film. Rhodium also has the advantages of high reflectance and high chemical stability. It should be noted that platinum is not the most expensive metal and is more common than rhodium.

**Figure 3:**
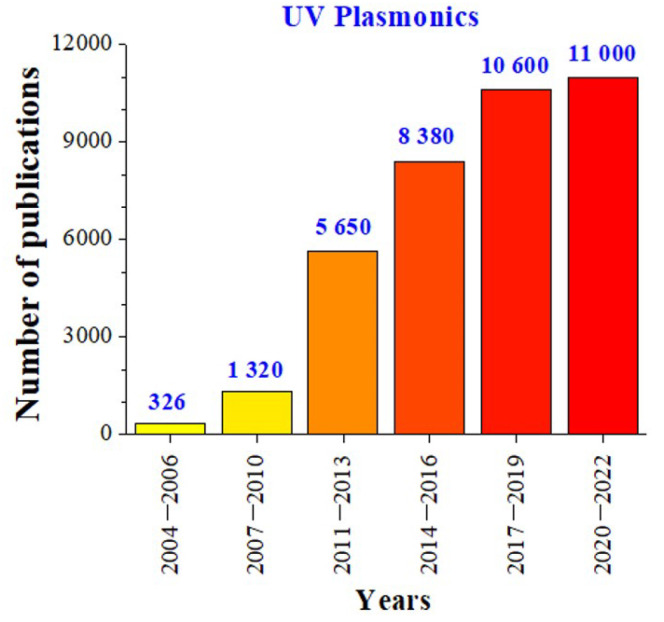
Recent research activity in “UV plasmonics of metal nanostructures”. The numbers of publications were determined by Google Scholar using the keyword “UV plasmonics”.

Biological substances, such as nucleic acids, DNA bases, and amino acids, have absorption bands in the UV region [72]. The tendency to study biological substances has led to huge scientific interest in UV plasmonics [65,73], which was confirmed by the recent increase in the number of publications (Figure 3).

#### 3.1.1. Rhodium Nanoparticle-Based Optical Sensing

Metallic nanoparticles have highly enhanced electric field near their sharp edges over a wide range of excitation wavelengths, which makes them suitable for creating sensitive plasmon-enhanced biosensors. Using fluorescence and Raman amplification with MEF and SERS methods makes it possible to reach detection limits as low as the single-molecule level [74,75]. Due to this feature, it is possible to develop hypersensitive biosensors based on MEF and SERS. In such methods, plasmonic metal nanostructures are required to amplify optical signals for biological detection.

Recently, research interest has focused on the development of SERS substrates. SERS is a powerful technique that increases the Raman signal from analytes on the metal surface. The Raman oscillations of molecules are generally not intense. Molecules near metallic nanoscale surfaces can show significantly enhanced Raman scattering intensity compared to individual molecules. SERS enhancements are attributed to two effects: (a) the electromagnetic effect, which is related to a huge increase in the local electromagnetic field near the metal surface [76], and (b) the chemical effect due to the resonant charge transfer between the molecules and the metal surface [77]. In this case, the chemical enhancement is due to an increase in the molecule polarizability. RhNPs have been used to study SERS [78]. Methylene blue (MB) was chosen to determine the amplification coefficients of RhNPs for the SERS experiment.

Rhodium multipods were used as SERS substrates [79]. Previous SERS studies on transition metals like Rh have not demonstrated intensive Raman enhancement [80]. Zettsu et al. compared the SERS activity of RhNP-film substrates prepared from three different Rh nanocrystals: (1) multipods synthesized in the presence of Ar, (2) multipods synthesized in air, and (3) nanocubes [79]. A study of nanocubes showed that these nanoparticles with an edge length of 9 nm showed an SPR peak at 250 nm. However, the absorption spectra of multipods with arm lengths of 11 nm showed SPR peaks at 360 nm (multipods synthesized in air) and 380 nm (multipods synthesized in Ar). It was found that both multipod film substrates with 4-mercaptopyridine (4-MP)-modified films gave more intensive Raman spectra, while RhNP substrates prepared from the nanocubes exhibited a rather weak Raman spectrum [79]. The multipods prepared in the presence of Ar showed the greatest SERS activity and showed 19 times stronger Raman signal than that of the nanocubes, indicating that the red-shift of the SPR peak for the multipods led to Raman enhancement [79]. The authors hypothesized that the sharp tips of the multipods provide additional amplification of the SERS signal [79].

Hunyadi Murph et al. studied SERS of 1.4 mM, 4-mercaptophenol (4-mPh) using bimetallic Ag–Rh nanoparticles [81]. The SERS spectra exhibited main peaks of 4-mPh. The Raman peaks of 4-mPh were detected only in the presence of bimetallic Rh–Ag nanostructures. The 4-mPh Raman peaks were also slightly blue-shifted compared to the native solution. Sangeetha et al. studied SERS of MB dye using Rh@DNA NPs with molar ratios of 0.08 M, 0.085 M, and 0.09 M [82]. In the absence of Rh@DNA NPs substrate, the Raman spectra of the probe molecule showed that only MB with a concentration of $10^{-3}$ M has weak Raman peaks at 445 cm${}^{-1}$, 1391 cm${}^{-1}$ and 1620 cm${}^{-1}$. In the presence of Rh@DNA NPs, the SERS signals from MB appeared at even lower concentrations down to $10^{-6}$ M. This proves the significant enhancement for the Raman bands at 445 cm${}^{-1}$, 1391 cm${}^{-1}$ and 1620 cm${}^{-1}$. The 0.08 M Rh@DNA NPs exhibited a maximum enhancement (EF) up to $10^{5}$ [82]. The same data were obtained from the SERS study by Kumaravel et al. [83]. In the near future, Rh@DNA NPs catalysts could be used in other photonics and electronics-related research. To investigate the MEF effect of the RhNPs, RhNP substrates of various densities were produced using electron beam deposition [84]. The constant MEF of the fluorophores was detected in the presence of RhNPs before and after autoclaving the substrates.

The most recent advance in RhNP-based optical sensing denotes to core-shell NP-based application [85]. The authors synthesized Au core–Rh shell nanoflowers (Au@Rh NPs) that are comprised of a spherical AuNP core and a shell containing Rh branches. They used such material as a model system to probe how the LSPR excitation from the AuNPs can lead to an enhancement in the catalytic activity of the Rh shells.

#### 3.1.2. Platinum Nanoparticle-Based Optical Sensing

Platinum nanoparticles (PtNPs) are increasingly used to enhance the capabilities of modern sensor technologies. The use of Pt nanostructures for the implementation of the UV–MEF method has been studied. Akbay et al. studied MEF of nucleic acids using platinum nanostructured substrates [86]. In the presence of Pt nanostructures, guanosine monophosphate exhibited a higher fluorescence intensity (about 20 times) compared to control samples on a quartz substrate. An optical sensor for determining oxygen concentration based on a Pt(II) complex and silver-coated SiO${}_{2}$ nanoparticles embedded in a sol–gel matrix was created [87].

The characteristics of SERS were studied using PtNPs of different morphologies obtained by chemical reduction [88] and physical ablation [89] methods. The surfaces of PtNPs for SERS application were stabilized by two types of capping agents (PVA and citrate) [90]. Rhodamine 6G dye was used to determine the effectiveness of various PtNPs for SERS. SERS spectra of 10 μM aqueous solutions of rhodamine 6G were obtained using different types of PtNPs as SERS substrates [90]. Raman enhancement was also demonstrated by nanoparticles obtained by chemical reduction methods. PVA-protected and uncoated nanoparticles led to weaker SERS effects with a lower signal-to-noise ratio.

The gap-enhanced Raman scattering characteristics of PtNPs were studied using 4-aminobenzenethiol (4-ABT) located at the gap between a flat Ag substrate and PtNPs that were 20–150 nm in size [91]. In the absence of PtNPs, the Raman peaks of 4-ABT placed on an Ag substrate were not identified. However, attachment of PtNPs to the protruding amino groups led to the ability to detect Raman peaks of the analyte. A higher SERS signal was obtained in the presence of larger PtNPs, regardless of the excitation wavelength.

**Figure 4:**
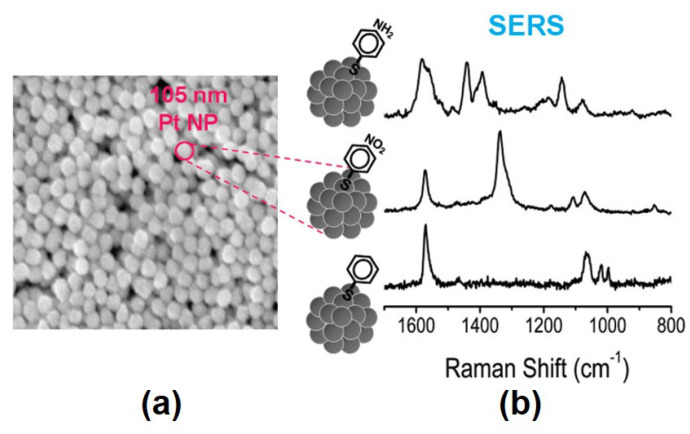
(**a**) FE-SEM image of the PtNP film. (**b**) SERS of 4-aminobenzenethiol (4-ABT), 4-nirobenzenethiol (4-NBT), and benzenethiol (BT) on the PtNP film measured at 488 nm excitation. Reprinted with permission from ref. [92]. Kim, K. et al., *Journal of Physical Chemistry C* **114**, 18679–18685 (2010). © 2010 American Chemical Society.

PtNPs with diameters of 28–105 nm were produced using a multi-stage seeded growth method. The absorption band of the PtNPs in the film state appeared at ∼330 nm regardless sizes of the PtNPs. A film self-assembled from larger Pt NPs had a higher absorption coefficient. The SERS parameters of their aggregates were investigated [92]. Figure 4 shows the SERS characteristics of three aromatic thiols, 4-nirobenzenethiol (4-NBT), 4-aminobenzenethiol (4-ABT), and benzenethiol (BT), on the PtNP film with an average particle diameter of 105-nm [92]. Higher-intensity Raman scattering was observed by shorter wavelength excitation for the adsorbates on a Pt film consisting of 105-nm PtNPs.

The syntheses of PtNPs of certain shapes, including a sphere, octahedron, octapod, and tetrapod, were carried out by varying the concentration of NaNO${}_{3}$ in a solvothermal process [93]. NaNO${}_{3}$ plays an important role in the synthesis of PtNPs of various shapes. These PtNPs were self-assembled on glass substrates to investigate the effect of the particle morphology on the Raman enhancement. 4-Mercaptopyridine was chosen as the analyte molecule. Pt tetrapods demonstrated improved SERS properties over other shaped particles. This effect is due to the enhanced local field effect around sharp corners and edges.

UV–SERS of adenine and $SCN^{-}$ were investigated by synthesizing various platinum and palladium nanoparticles, namely, Pt nanospheres, Pt nanocubes, and Au@Pt or Au@Pd nanoparticles with different shell thicknesses [94]. These researchers investigated the crystal quality-dependent UV–SERS activity. High-quality nanocrystals are required for higher amplification, but low-quality nanocrystals may not be effective for SERS [94].

One study described a method for obtaining structured Pt films, which were used to create SERS-active substrates with a significant Raman enhancement from benzenethiol [95]. Structured surfaces were uniform, smooth, and stable. The advantage of structured surfaces is that the performances of the SERS substrates can be adjusted by varying both the template sphere diameter and the film thickness [95]. In addition, these surfaces can be reused after cleaning in some cases.

Four different types of PtNPs with sizes of 29, 48, 73, and 107 nm were investigated as potential UV–SERS substrates for melamine detection [96]. The absorption bands of all these PtNPs are in the UV range (about 200 nm). A 244 nm laser beam was used as an excitation light for the UV–SERS experiments. The 29 nm PtNPs demonstrated the highest SERS activity. PtNPs with sizes of 48–73 nm show approximately the same increase in SERS intensity. The 107 nm PtNPs showed the lowest SERS activity.

The potential of the Pt nanostructures for SERS applications was evaluated using rhodamine 6G as the probe molecule [97]. Almost all Raman modes were observed when using the Pt nanostructure. However, a silicon substrate without a Pt nanostructure did not show any noticeable Raman signal.

The most recent advance in Pt-based optical sensing consists of the creation of core-shell nanoparticles and 2D surfaces. Fan et al. synthesized Au-core, Pt-shell (Ag@Pt) NPs and used them for plasmonic catalysis [98]. Bimetallic nanocatalysts from two metal elements have been used as a promising method for high catalytic performance based on synergistic effects. Pang et al. used Ag@Pt NPs as an enzymatic reporter to identify microcystin-leucine arginine antibodies [99]. Proniewicz et al. used SERS and tip-enhanced Raman scattering (TERS) to characterize the selective adsorption of phenylboronic acid phosphonic acid (PBA–PA) derivatives on the surface of PtNPs from an aqueous solution and from air [100]. Lin et al. presented a SERS-based nanoprobe (Au@Pt core-shell NPs) for direct and simultaneous identification of multiple mitochondrial reactive oxygen species by their distinct Raman fingerprints without introduction of Raman reporters [101].

In Table 1, we compared representative results for the synthesis methods and optical sensing applications of rhodium- and platinum-based nanomaterials.

### 3.2. Gold- and Silver-Nanoparticle-Based Optical Sensing

#### 3.2.1. Gold Nanowire-Based Optical Sensing

**Figure 5:**
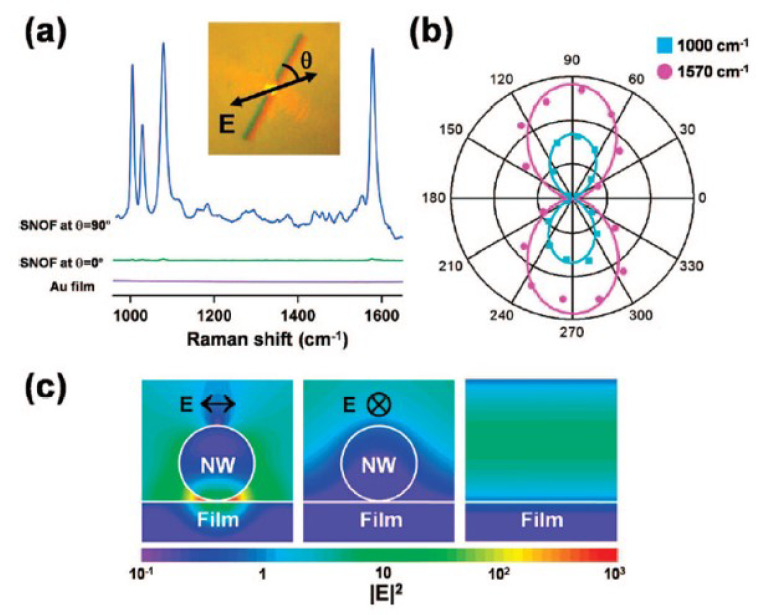
(**a**) Polarization-dependent SERS spectra of benzenethiol (BT) for the single gold nanowire on the gold film. The traces with the excitation light polarization (blue) perpendicular and (green) parallel to the nanowire were measured at the same location on the nanowire. The violet trace was measured for the flat Au film as a control. (**b**) Polar plots of the integrated intensities of the (cyan square) 1000 cm${}^{-1}$ and (magenta circle) 1570 cm${}^{-1}$ Raman bands of BT with respect to θ (the angle between the excitation light polarization and the Au nanowire axis). (**c**) Calculated distributions of the local electric field intensities, ${\left|E\right|}^{2}$, for the Au nanowire on the Au film with the excitation light polarization (left) perpendicular and (middle) parallel to the nanowire axis and (right) for the smooth Au film. Reprinted with permission from ref. [102]. Yoon, I. et al., *Journal of the American Chemical Society* **131**, 758 (2009). © 2009 American Chemical Society.

Yoon et al. studied SERS of benzenethiol (BT) by a single gold nanowire on a gold film (Au/Au SNOF) [102]. Gold nanowires were synthesized on a sapphire substrate in a horizontal quartz tube furnace system using a vapor transport method. Figure 5 shows the polarization-dependent SERS spectra of BT adsorbed on the Au/Au SNOF [102]. The gap-enhanced Raman scattering signal was observed at the gap between the Au nanowire and the Au film when perpendicularly polarized excitation light (blue) was incident on the Au nanowire, but this enhancement was not observed by parallel-polarized excitation light (green). In the absence of Au nanowire or Au film, the Raman peaks of BT were not observed. These results confirm that the gap-enhanced Raman scattering signals were induced from the local field enhancement due to the LSPR. For comparison of experimental results with theoretical expectations, they calculated the spatial intensity distribution near the Au nanowire using the three-dimensional FDTD method. FDTD calculations confirm that the gap-enhanced Raman scattering signals were observed at the gap between the Au nanowire and the Au film for transverse plasmon mode excitation.

Ranjan et al. studied anisotropic SERS of nanowires and nanoparticle arrays [103]. In longitudinal plasmon mode excitation, gold nanowires behave like bulk metal, while in transverse plasmon mode excitation, the local field enhancement occurs due to LSPR. Meanwhile, for nanoparticle arrays, higher SERS intensity is found along the particle chain. The SERS intensity for light polarized along the particle chain is much higher than the intensity for light polarized in the perpendicular direction.

#### 3.2.2. Gold Nanoparticle-Based Optical Sensing

Nam’s group studied SERS using DNA-tailorable AuNPs [104]. The SERS intensity is proportional to the probe concentration, and SERS showed a limit of detection down to a probe concentration of 10 fM. The nanogap-enhanced Raman scattering intensity had an enhancement factor higher than $1.0\times 10^{8}$, which was sufficient for single-molecule detection. In these studies, the Raman enhancement was attributed to the plasmon resonance effect of Au nanowires. They reported that the Raman enhancement factors were on the order of $10^{14}$ to $10^{15}$ for a single rhodamine 6G molecule adsorbed on the selected AgNPs [105]. Kneipp’s group studied SERS in single molecule and single living cell using AuNPs [106,107].

**Figure 6:**
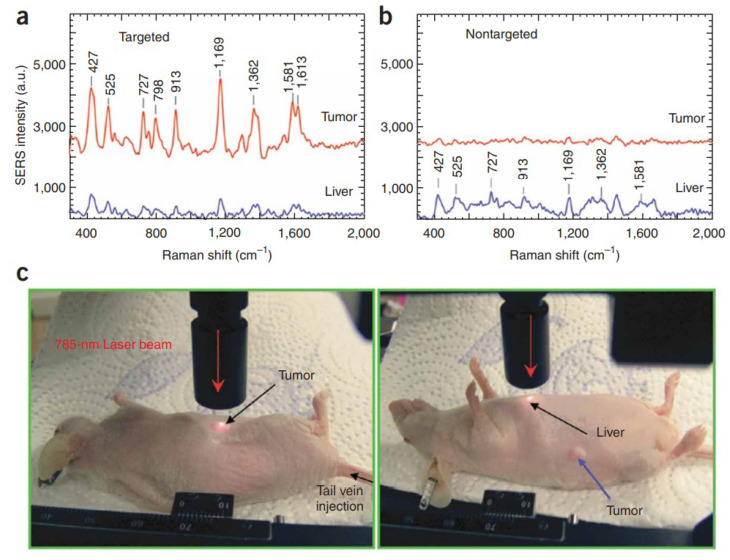
(**a**,**b**) SERS spectra of the tumor and the liver obtained using (**a**) targeted and (**b**) nontargeted gold nanoparticles. (**c**) Photographs of a 785-nm laser beam focused on the tumor sites or on the anatomical location of the liver. The spectra were background subtracted and shifted for better visualization. Reprinted with permission from ref. [108]. Qian, X., Peng, X. H., Ansari, D. et al. *Nature Biotechnology*, **26**, 83–90 (2008). © 2008 Springer Nature.

The plasmon-enhanced SERS activity of AuNPs can be applied to distinguish between normal cells and cancerous cells. Qian et al. studied in vivo tumor targeting and SERS detection using single-chain variable fragment (scFv)-conjugated AuNPs [108]. The scFv antibody-conjugated AuNPs can identify the epidermal growth factor receptor (EGFR) of tumor cells. Figure 6a,b show the SERS spectra of (red) the tumor site and (blue) the liver site obtained using a 785 nm laser as an excitation light. The SERS spectra were obtained 5 h after nanoparticle injection. The power of excitation light and the Raman signal integration time were 20 mW and 2 s, respectively. The Raman reporter molecule was malachite green. SERS signals in the tumor region were significantly enhanced for the targeted nanoparticles, indicating that the scFv-conjugated AuNPs were able to detect EGFR-positive tumors in vivo [108].

#### 3.2.3. Silver Nanoparticle-Based Optical Sensing

Rycenga et al. demonstrated synthesis methods, control factors, and optical sensing applications (SERS; SEF; control of light with plasmonic antennas) of AgNPs [109]. He et al. deposited an AgNP monolayer on a (3-aminopropyl) triethoxysilane (APTES)-functionalized glass slide [110]. Their SERS performance was studied using rhodamine 6G as a target analyte. These authors studied the effect of AgNP size on SERS signal enhancement. Moskovits’ group examined SERS using Ag nanowire bundles as an efficient SERS platform [111].

Nie and Emory studied single-molecule detection of rhodamine 6G using AgNP-based SERS [105]. Fan et al. studied quasi single-molecule detection by AgNPs self-assembled on a 3-mercaptopropyl trimethoxysilane (MPTMS)-functionalized glass slide [112]. Nile blue A and oxazine 720 were used as probe molecules.

Gopal et al. evaluated the freshness of fruits and vegetables using SERS of AgNPs and AuNPs supported on graphene nanosheets [113]. Fruits and vegetables such as wax apple, lemon, tomato, red pepper, and carrot were investigated. These authors used graphene-enhanced Raman spectroscopy as a non-destructive tool for diagnosis of the freshness of fruits and vegetables.

In Table 2, we compared representative results for the synthesis methods and optical sensing applications of the gold- and silver-based nanomaterials.

#### 3.2.4. Practical Applications of Plasmonic Metal Nanoparticle-Based Optical Sensing

Craig et al. demonstrated the use of SERS as a reliable tool for the discrimination of food-borne pathogenic microorganisms, which include detection of bacteria, spores, and viruses [121]. Sing et al. reviewed applications of metal nanoparticles in the fields of food technology, food packaging, and food security [122]. Terry et al. described the applications of SERS in environmental contaminant monitoring [123]. The authors explored methods for the SERS detection of inorganic, organic, and biological contaminants including heavy metals, plastic particles, dyes, pharmaceuticals, pesticides, viruses, bacteria, and mycotoxins.

Recently, there have been many reports on plasmonic metasurfaces that use self-assembly or arbitrary patterns of metal nanoparticles [124,125,126,127]. Metasurfaces can control light propagation by changing their phase, amplitude, polarization, or spectrum. Plasmonic metasurfaces can be employed in various applications such as broad band absorber /reflector, meta lens, hologram, nanoantennas, photovoltaics, surface-enhanced fluorescence, SERS, and biosensing.

## 4. Synthesis of Rhodium and Platinum Nanoparticles

Generally, the nanoscale fabrication methods are divided into two major categories, i.e., ”top–down” and “bottom–up” methods according to the processes involved in creating nanoscale structures. Top–down approaches create nanoscaled structures by controlled removal of materials from larger or bulk solids [128]. By contrast, bottom–up approaches build molecular or atomic components into nanoscale assemblies based on complex mechanisms and technologies [128]. Experimentally, metal nanoparticles can be fabricated by chemical, physical, and green synthesis methods, as can be seen from Table 3 [129,130,131,132]. The physical method is a top-down approach. On the other hand, chemical and biological methods use a bottom-up approach. The creation of metal nanoparticles with controlled morphologies has attracted increasing research interest as these have technological potential in photonics, sensors, and biomedicine due to their unique physicochemical properties [133,134].

### 4.1. Chemical Synthesis of Rhodium and Platinum Nanoparticles

The chemical methods for the synthesis of Rh and Pt nanoparticles are based on reduction from metal salts. Synthesis, growth, and shape of nanoparticles are regulated by changing the concentration of the reductant, stabilizer, pH of the medium, and temperature. Such a method involves the use of precursor salts for their reduction to metal monomers. Then, the nucleation process starts. Growth of nanoparticles automatically stops when they formed a cluster of reduced metal atoms. Thus, growth is controlled by the reducing agent. In this way, the particles reach a stable size and shape.

Various chemical synthesis methods can be used for the synthesis of metal nanoparticles. Various compositions and reaction conditions are used for synthesis. The advantages of chemical methods are cost-effectiveness, easy processing, high yield, particle size control, thermal stability, and homogeneous dispersion.

#### 4.1.1. Chemical Synthesis of Rhodium Nanoparticles

**Figure 7:**
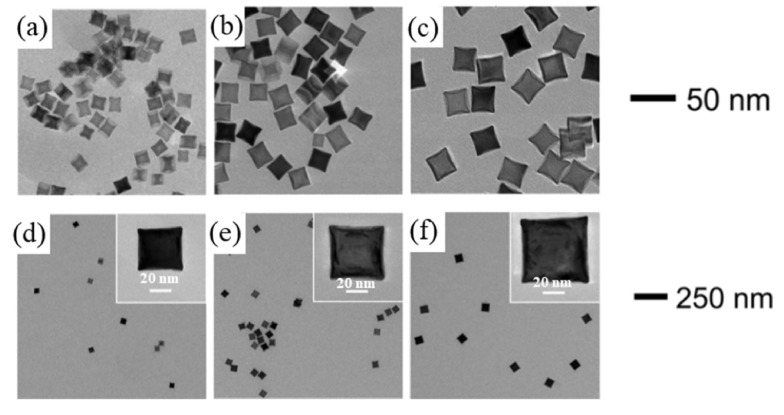
TEM images of Rh nanoparticles from the slow-injection methods, with corresponding average edge lengths of (**a**) 15 nm, (**b**) 21, (**c**) 27 nm, (**d**) 39 nm, (**e**) 47 nm, and (**f**) 59 nm. Reprinted with permission from ref. [135]. Xu, L. et al., *Heliyon* **5**, e01165 (2019). © 2019 Elsevier Ltd.

It is possible to create the most suitable nanostructures for specific scientific tasks by changing their shape and size during synthesis process. In one report, methods for the synthesis of RhNPs based on wet chemistry were identified, including synthetic approaches and special means of controlling the shape and size [135] (Figure 7). Toshima and colleagues first announced their study of Rh nanocrystals in the late 1980s [136]. Later, the polyol synthesis of branched Rh nanocrystals was described [79]. The Rh nanocrystals were produced by the reduction of Na${}_{3}$RhCl${}_{6}$ in ethylene glycol.

Lokesh et al. demonstrated a one-step synthesis route for the fabrication of RhNPs, where a macrocyclic complex of cobalt aminophthalocyanine was used as a stabilizer [137]. Transmission electron microscopy (TEM) studies have shown that the nanoparticles have a spherical shape with an average diameter of 3 to 5 nm. In the modified polyol method, PVP-stabilized RhNPs were produced by the reduction of RhCl${}_{3}$ in ethylene glycol at 160 °C using AgNO${}_{3}$ [138].

Bimetallic Rh–Pt nanoparticles [139] were created to control catalytic CO oxidation. The composition of bimetallic Rh–Pt nanoparticles was changed at a particle size of ∼9 nm. AgNO${}_{3}$ was used for the controlled synthesis. There are different types of morphologies for such nanoparticles including cubes, octahedrons, cuboctahedrons, tetrahedra, and other regular and irregular forms. The shapes of the nanoparticles can be controlled during the reduction of RhCl${}_{3}$ in ethylene glycol and PVP. In general, the mechanisms of their seed creation and growth are described by the chemical reduction of metal salts or the chemical decomposition of metal complexes with the formation of metal atoms [140]. A method for the synthesis of Rh icosahedrons with sizes up to 12.0 ± 0.8 nm was demonstrated using $Rh{\left(acac\right)}_{3}$ as a precursor, PVP (molecular weight ≈ 40,000) as a reducing agent, and benzyl alcohol as a colloidal stabilizer [141].

RhNP fabrication by chemical reduction using NaBH${}_{4}$ as a reducing agent was proposed [82]. The prepared Rh@DNA NPs had an average particle size of ∼5 nm and was employed for catalysis and SERS experiments.

RhNPs can also be synthesized by the slow-injection polyol method. Unseeded (pre-synthesized Rh seeds were not added) and seeded (pre-synthesized Rh seeds were added) slow-injection methods were used for the synthesis of monodispersed Rh nanocubes [142]. Briefly, a solution of KBr in ethylene glycol was kept at 160 °C, and then solutions of RhCl${}_{3}$ and PVP were injected into it separately at low speed using a dual-channel syringe pump. Controlled nanocube size and LSPR wavelength in the UV region were obtained.

Rh, Ag, and Rh–Ag nanoparticles were fabricated using the polyol method [81]. Such nanomaterials have been obtained by the co-reduction of metal precursors with polyol at elevated temperature. Careful selection of the ratio between the reactants and the reaction temperature can be used to regulate the size and morphology of the nanomaterial. Rh–Ag nanoparticles have a plasmonic band at about 410 nm. It differs from the SPR band expected from individual monometallic colloids. This spectral shift of the plasmon resonance wavelength indicates that the resulting nanomaterials are bimetallic.

For UV plasmonics, flat tripods of 8-nm RhNPs had an LSPR band at about 330 nm when synthesized by a modified polyol reduction method [143]. Raman and fluorescence enhancement were detected upon attachment of p-aminothiophenol (PATP). The fluorescence intensity increased by charge transfer. The local field enhancement and charge transfer represent important steps towards UV plasmonics and photocatalytic applications. Decahedral Rh nanocrystals have been synthesized with a purity of almost 90% by systematically adjusting the concentration of the metal precursor Rh(acac)${}_{3}$, the molecular weight and amount of the poly(vinyl pyrrolidone), and the chain length of polyol to favorably control the homogeneous nucleation process [144]. Lee et al. described a one-pot method based on polyol reduction for the easy synthesis of high-purity decahedral Rh nanocrystals with sizes less than 20 nm [144]. The nucleation and growth processes for synthesizing colloidal metal nanoparticles of various forms have also been studied [145].

#### 4.1.2. Chemical Synthesis of Platinum Nanoparticles

The simplest way to obtain PtNPs is to gradually add chloroplatinic acid (H${}_{2}$PtCl${}_{6}$) to poly(vinyl pyrrolidone)–ethylene glycol (PVP–EG) while heating to 120 °C. Nanoparticles with sizes of 3–8 nm were obtained by adjusting the amount of Pt precursor [146]. Further modifications of the method made it possible to control the morphology of nanoparticles. Thus, Koebel et al. obtained tetrahedral and octahedral nanoparticles in ethylene glycol using PVP as a stabilizer. Sodium nitrate was also used to obtain this particle shape [147].

**Figure 8:**
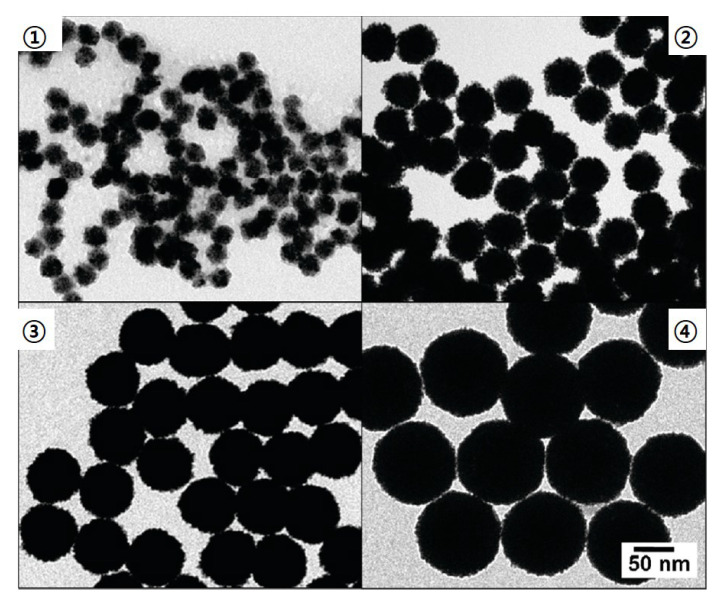
TEM image of monodispersed Pt nanospheres with average diameters of 29–107 nm and extremely small standard deviations of 3%. ➀ 29 nm, ➁ 48 nm, ➂ 73 nm, and ➃ 107 nm. [88]. Reprinted with permission from ref. [88]. Bigall, N. C. et al., *Nano Letters* **8**, 4588 (2008). © 2008 American Chemical Society.

A simple synthesis method of the monodispersed Pt spheres with diameters up to 107 nm and extremely small standard deviations of 3% was presented [88] (Figure 8). The reaction proceeds in an aqueous solution using a multi-stage seeded approach. Long et al. successfully synthesized PtNPs in the form of nanocubes and nanooctahedra with controlled sizes of 5–7 and 8–12 nm, respectively [148]. An advanced polyol method was performed by adding AgNO${}_{3}$, and the molar ratio of AgNO${}_{3}$ and H${}_{2}$PtCl${}_{6}$ solutions was changed to synthesize PtNPs with controlled morphology.

PtNPs were produced by heating an aqueous solution containing H${}_{2}$PtCl${}_{6}$, polyvinyl acetate, and polyvinyl alcohol [149]. A heat treatment method produced stable, polymer-protected PtNPs of 2–7 nm in diameter. Here, polyvinyl acetate serves as both a reducing agent and a protective agent. In addition to the preparation of PtNPs by the polyol method, methods to synthesize PtNPs by phase condensation of sprayed atomic vapor in an inert gas [150] and galvanic displacement [151] were also used.

### 4.2. Physical Synthesis of Rhodium and Platinum Nanoparticles

For biophysical, biomedical, and catalytic applications, the chemically synthesized nanoparticles require additional purification processes to remove excess surfactants and residual reagents. In this case, it is necessary to use other methods for the synthesis of nanoparticles.

Pulsed laser ablation in liquid without any chemical reagents has attracted interest from researchers for the fabrication of pure noble metal nanoparticles for biomedicine and catalysis [152]. Liquid laser ablation for the production of metal nanoparticles is based on the ejection of nanoparticles by a laser pulse that irradiates a solid metal target immersed in water or an aqueous solution. Laser interactions with matter and subsequent ablation strongly depend on laser beam characteristics (laser power, energy density, pulse duration, and laser focusing conditions), target parameters (shape, density, and compactness), and the background liquid conditions (temperature and pH).

Laser synthesis of metal nanoparticles using high-power lasers is a chemically clean process and is attractive for the industrial production of functional nanomaterials [153]. The creation of nanoparticles using laser ablation still has some challenges, such as the production of nanoparticles of certain morphologies, low polydispersity, and low productivity [154].

#### 4.2.1. Physical Synthesis of Rhodium Nanoparticles

RhNPs have been produced by laser ablation of a Rh target dipped in pure water (W–Rh–NPs) or ethanol (E–Rh–NPs) [155]. Colloidal RhNPs were obtained according to a typical liquid laser ablation procedure. During synthesis, a 1064 nm laser beam with a pulse width of 6 ns and an energy density of 7.6 J/cm${}^{2}$ is focused on a bulk Rh plate dipped in water or ethanol. In both cases, the nanoparticles had regular spherical shapes. The average diameters of RhNPs were 28 ± 14 nm for W–Rh–NPs and 14 ± 11 nm for E–Rh–NPs. A smaller average size was also observed during 532 nm laser ablation [156]. The 532 nm laser beam used to ablate the target can also be absorbed by previously produced nanoparticles. Nanoparticles are dispersed in the liquid layer above the target, resulting in a smaller nanoparticles than those made using NIR pulses.

Kishida et al. demonstrated that monodispersed shelled RhNPs were obtained by hydrolysis of tetraethylorthosilicate in the presence of Rh precursors followed by heat treatment [157]. As a result, microemulsions of RhNPs coated with silica were obtained. Such microemulsions include 4-nm RhNPs located in the center of a silica particle.

Size-selective RhNPs were first synthesized using a simple UV-light irradiation pathway [78]. The synthesis was carried out by reduction of RhCl${}_{3}$ with alkaline 2,7-dihydroxy naphthalene (DHN) on DNA scaffolds for 5 h at room temperature and under ambient conditions.

#### 4.2.2. Physical Synthesis of Platinum Nanoparticles

Physical methods for the synthesis of PtNPs include ball milling, ion sputtering, physical vapor deposition, and laser ablation [151,158,159]. For example, it was reported that PtNP layers with a thickness of 2.5–3.3 nm formed over the alumina-coated silicon carbide ($Al_{2}O_{3}$@SiC) rectangular plates by heating chloroplatinic acid in a muffle furnace at a temperature of 350 °C [160]. Particles of 8–9 nm in size were obtained using the laser ablation with a neodymium-yttrium-aluminum garnet (Nd–YAG) laser [161].

Polydispersed colloidal PtNPs of various sizes were obtained by ablation of a Pt target using a nanosecond laser [162]. Other lasers (266, 532 and 1064-nm) were employed for the ablation experiment, and several stabilizers were also used, namely, citrates and PVP, PEG, and PVA polymers. Visible and IR laser ablation led to the formation of amorphous PtNP spheres. The diameter distribution of these particles had a maximum in the range of 5–10 nm and also had a shoulder extending up to 25 nm. Small crystalline nanoparticles (1–4 nm in diameter) and larger particles (6–8 nm) were obtained using 266 nm laser ablation, which is described here for the first time.

Mafune et al. synthesized PtNPs by laser ablation of a Pt metal plate in an aqueous solution of sodium dodecyl sulfate (SDS) [89]. The obtained nanoparticles have particle sizes of 1–7 nm in diameter. It has been established that stable PtNPs can be obtained by this method even in pure water. The absorption spectra of PtNPs did not practically differ from those prepared by a chemical method. Mafune et al. reported that 6 nm PtNPs were obtained by 355 nm laser ablation of the Pt precursor solution at a given concentration of SDS [163]. In addition, 5–20 nm PtNPs were obtained using a physical-vapor-deposition coating at a temperature of 700 °C [164].

PtNPs were synthesized by plasma treatment of Pt wire in pure water. Plasma-induced electrons and radicals bombarded the Pt surface, releasing Pt atoms, affording highly dispersed PtNPs with a size of 2 nm [165].

### 4.3. Green Synthesis of Rhodium and Platinum Nanoparticles

**Figure 9:**
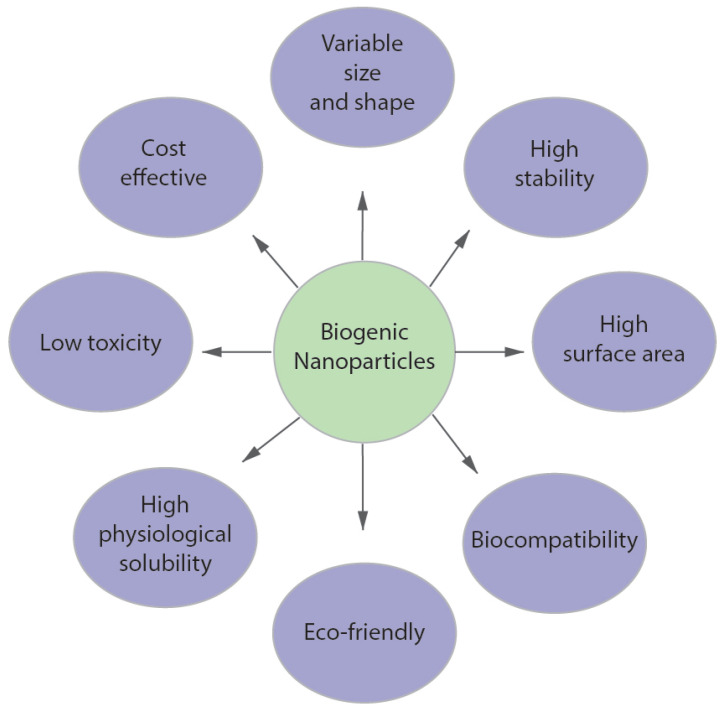
Advantages of biogenic synthesis over chemical and physical synthesis of metal nanoparticles.

Biogenic nanoparticle synthesis has attracted great interest due to its biocompatibility and potential applications in energy harvesting, catalysts, antimicrobial agents, gene therapy, and sensors. Diverse biomolecules and microorganisms have been used as templates to create nanoscale materials. Biogenic nanoparticle synthesis has several advantages. For example, this method is easy, cost-effective and reduces the chemical load on the environment [166,167,168] (Figure 9). Various types of microorganisms, including fungi, bacteria, and yeast, can reduce metal salts to nanoparticles [132,168,169]. In the case of green synthesis, it is possible to replace expensive chemical reducing agents with biomolecules. Thus, nanoparticles synthesized by a biological method are cheaper than nanoparticles synthesized by physical and chemical methods (Figure 9). The lipid bilayer contained in many biogenic nanoparticles makes such nanoparticles more stable and provides better physical solubility. Such nanoparticles are more suitable for biomedical applications. In addition, the morphologies of nanoparticles can be regulated by changing various reaction parameters, such as pH, substrate availability, and reaction time [170]. Thus, biogenic nanoparticle synthesis has become an environmentally friendly, economical alternative to physical and chemical methods for the synthesis of metal nanoparticles.

#### 4.3.1. Green Synthesis of Rhodium Nanoparticles

Tamaoki et al. demonstrated a biogenic nanoparticle synthesis method using *Shewanella* SM1 algae, which reduces metal ions [171]. The RhNPs were deposited intracellularly in the *S. algae*. Biogenic synthesis of Rh, Ag, Pd, Fe, Ni, Co, Ru, Pt, and Li nanoparticles at room temperature was performed using *Pseudomonas aeruginosa* SM1 without the addition of nanoparticle stabilizers or the control of pH and temperature [172].

#### 4.3.2. Green Synthesis of Platinum Nanoparticles

Biological methods of synthesizing nanoparticles involve several microorganisms, such as algae, fungi, bacteria, and actinomycetes. Thus, researchers are interested in plant-extract-based nanoparticle synthesis, which is easier to use. An extract of the herb *Fumariae Dobrucka* was used to obtain pentagonal and hexagonal forms of PtNPs [173]. The synthesis was carried out at 50 °C for 4 h. A color change from yellow to brown was observed. Advanced analytical methods such as TEM, SEM, atomic force microscopy (AFM), and Fourier-transform infrared spectroscopy (FT–IR) were used for investigation of the morphologies of the nanoparticles. The obtained nanoparticles were irregular rods about 4 nm in size. There were also clustered nanoparticles about 10 nm in size. PtNPs have also been synthesized using *Azadirachta indica* [174]. TEM images showed the formation of spherical nanoparticles with sizes of 5–50 nm.

Date extracts were used for the biogenic synthesis of PtNPs [175]. The obtained sizes of PtNPs varied from 1.3 to 6 nm, and nanoparticles were uniform small spheres. Flavanols were employed as reducing and blocking agents. The obtained nanoparticles exhibited antibacterial effects against *E. coli* and *B. subtilis*. Leaf extracts of *Lantana camara* L. were used to prepare PtNPs [176]. It was determined that the nanoparticles were spherical in shape and 35 nm in size. The synthesis of spherical PtNPs using *Prunus yedoensis* gum extract was also described [177]. The obtained nanoparticles demonstrated efficacy against pathogenic fungi. The synthesis of PtNPs from the leaf extract of *Barleria prionitis* has been described [178]. *B. prionitis* extract was used to obtain monodispersed nanoparticles with sizes of 1–2 nm. The synthesized PtNPs demonstrated inhibitory effects on MCF-7 breast cancer cells.

There are many scientific works describing platinum nanoparticle synthesis using various plant extracts, such as *Cochlospermum gossypium* [179], *Anacardium occidentale* [180], *Diopyros kaki* [181], *Cacumen platycladi* [182], and *Punica granatum* [183]. Despite these significant achievements, green synthesis of PtNPs has not become widespread.

## 5. Synthesis of Gold and Silver Nanoparticles

AuNPs and AgNPs have produced by various methods, such as thermal evaporation [184,185], spray pyrolysis [186], laser ablation [187], citrate reduction of metal precursor salts [188,189,190], chemical bath deposition [191], hydrothermal synthesis [192], reductions of metal precursor salts with microwave [193], gamma-ray [194] and plasma [195,196,197].

Among these methods, the citrate reduction method (called as Turkevich method) has been extensively employed due to its simplicity, high yield, homogeneous dispersion, and low cost [188,189,197,198,199,200]. After gold precursor HAuCl${}_{4}$ was reduced by the citrate reduction at boiling temperature, the color of the gold precursor solution changed from colorless to red due to reduction of gold ions to zero-valent gold nanoparticles [198,199,200,201]. Addition of sodium borohydride (NaBH${}_{4}$) to the Turkevich method can simplify the synthesis of metal nanoparticles by eliminating the heating process [200,202,203]. Seney et al. synthesized silver nanoparticles by aqueous reduction of AgNO${}_{3}$ with NaBH${}_{4}$ and investigated SERS activity of silver nanoparticles using *trans*-1,2-Bis(4-pyridyl)ethylene (BPE) as the Raman label [204].

**Figure 10:**
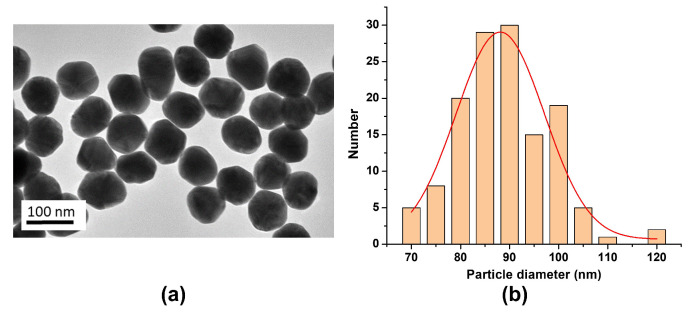
(**a**) TEM image and (**b**) particle size distribution of gold nanoparticles prepared by the citrate reduction method. Reprinted with permission from ref. [201]. Lee, G. J. et al., *Journal of Physics D: Applied Physics* **53**, 095102 (2019). © 2019 IOP Publishing.

Lee et al. demonstrated that the AuNPs were successfully obtained by the citrate reduction method (Figure 10) [201]. The obtained AuNPs featured a monodispersed spherical shape with an average diameter of 89 nm. Their absorption spectra showed the surface plasmon resonance at wavelength of 550 nm. These authors demonstrated SERS sensing of chiral-achiral polymer blends by AuNPs and investigated the SERS and their chiroptical characteristics using surface plasmon resonance effects of gold nanoparticles [201].

**Figure 11:**
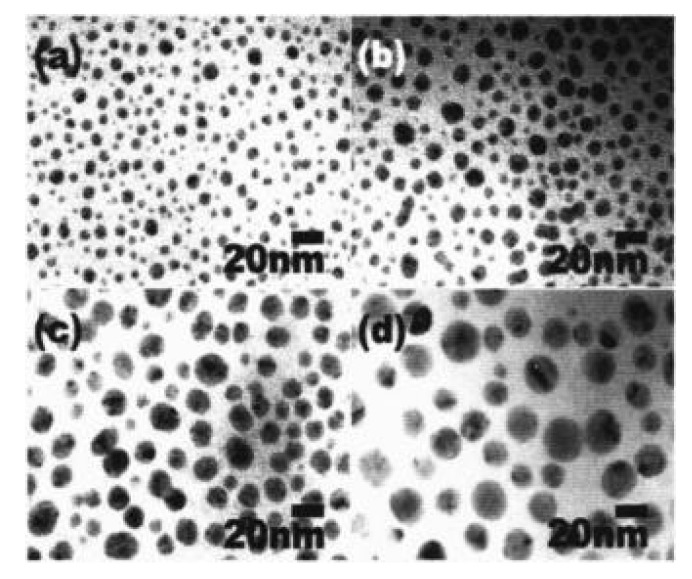
TEM images of silver nanoparticles prepared by curing PI /Ag /PI samples: (**a**) 2.5, (**b**) 5, (**c**) 10, and (**d**) 15 nm Ag film. Reprinted with permission of AIP Publishing from ref. [184]. Lim, S. K. et al., *Journal of Applied Physics* **98**, 084309 (2005). © 2005 AIP Publishing.

Meanwhile, metal nanoparticle films can be fabricated by different methods, such as dip coating [205], and thermal evaporation [184,185]. Lim et al. reported that silver nanoparticles were produced by sandwiching a thin silver film between two polyimide (PI) precursor layers and then curing PI /Ag /PI samples at 400 °C for 1 h under vacuum (Figure 11) [184]. Szunerits et al. fabricated gold nanoisland films on indium tin oxide by thermal evaporation of 2–6 nm gold films and thermal annealing (12 h at 150 °C or 1 min at 500 °C) [206]. These authors studied LSPR sensing performance of gold nanoislands for biotinylated bovine serum albumin (biotin-BSA).

Billot et al. fabricated gold nanostructures of desired shape, size, and arrangement on glass substrates through electron beam lithography (EBL) and lift-off techniques [207] They studied the SERS efficiency of gold nanowires arrays for the probe molecule, trans-1,2-bis(4-pyridyl)ethylene (BPE) using the 632.8 nm line of a He–Ne laser as an excitation light. Tan et al. fabricated periodic arrays of gold nanoparticles by spin coating of polystyrene nanospheres, electron beam deposition of gold films, nanosphere lithography, and thermal annealing [208].

**Figure 12:**
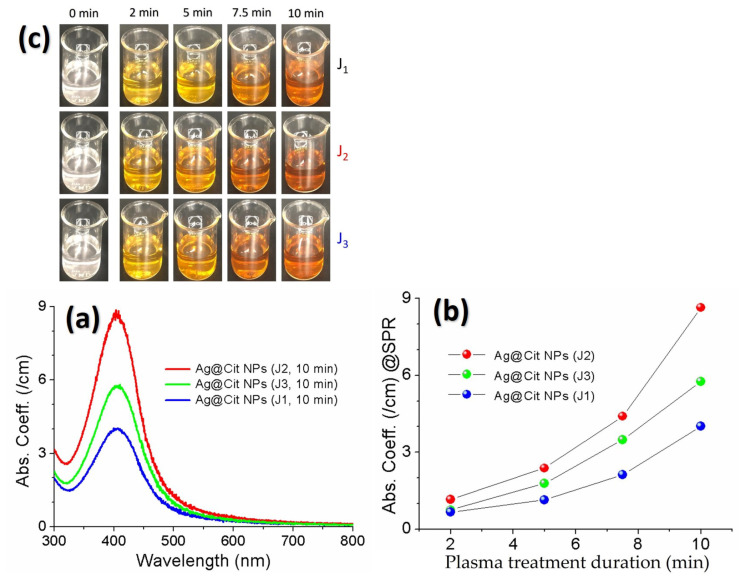
(**a**) Absorption spectra of the silver nanoparticle solutions obtained by treating $AgNO_{3}$–TSC–DW with the J1, J2, and J3 jets. (**b**) SPR peak intensity of the silver nanoparticle solutions as a function of the plasma treatment duration. (**c**) Color change of the silver nanoparticle solutions over the plasma treatment duration to 10 min. Reprinted with permission from ref. [211]. Acharya, T. R. et al., *Nanomaterials* **12**, 2367 (2022). © 2022 MDPI.

Microwaves [193], gamma-rays [194], pulsed lasers [209], and plasmas [195,196,197] have been used for the physical synthesis of metal nanoparticles because they can be operated with smaller amounts of chemicals and shorter processing time compared with those of chemical synthesis. The plasma synthesis of metal nanoparticles is relatively simple, inexpensive, and efficient. In particular, atmospheric-pressure plasma jets (APPJs) have generated much interest as a promising method for synthesizing metal nanomaterials [195,210]. APPJs can synthesize metal nanoparticles in a shorter processing time. Figure 12a shows the absorption spectra of the AgNP solutions produced by the APPJs with plasma-plume-lengths of 2.5, 8.5, and 12.5 cm [211]. The creation of silver nanoparticles was confirmed by a color change from colorless to dark yellow (Figure 12c). A larger amount of silver nanoparticles was obtained with a longer plasma treatment duration. For a plasma treatment duration of 10 min, absorption coefficients of silver nanoparticles at the SPR peaks were 4.0/cm, 8.6/cm, and 5.8/cm for the J1, J2 and J3 jets, respectively (Figure 12b).

Nikolov et al. created Ag and Au nanoparticles by pulsed laser ablation of Ag and Au targets, immersed in double-distilled water [209]. The targets were irradiated for 20 min by 15 ns laser pulses at the fundamental (λ = 1064 nm) and the second harmonic (SHG) (λ = 532 nm) wavelengths of a Nd–YAG laser. Yin et al. synthesized large-scale and size-controlled silver nanoparticles by treating an aqueous solution of silver nitrate and trisodium citrate with microwaves [193]. Formaldehyde was used as a stabilizer and a reductant. Eisa et al. generated polyvinyl alcohol (PVA)/silver nanocomposites by the reduction of silver ions with gamma-irradiation [194]. These authors reported that monodispersed silver nanoparticles are embedded homogenously in a PVA matrix and that the obtained nanoparticles exhibited a uniform shape and a very narrow size distribution with an average size of 17 nm.

## 6. Concluding Remarks

In this review, we described fundamental concepts, optical sensing applications, and synthesis methods of plasmonic metal nanoparticles. We presented a brief overview of the existing chemical, physical, and green methods for the synthesis of Rh, Pt, Au, and Ag nanoparticles. Optical sensing applications of Rh and Pt nanoparticles by UV plasmonics such as UV–MEF and UV–SERS were described. Au and Ag nanoparticles have received a lot of interest due to various applications, such as optical sensors, chemical sensors, biosensors, food and health safety monitoring, pathogen detection, cancer diagnostics, and biomedicine. These nanoparticles are the most sensitive for detecting a wide range of analytes from ions, biomolecules, macromolecules, and microorganisms. They provide a promising platform for highly sensitive detection of analyte materials and cancer cells. Optical sensing and biosensing applications are based on the plasmonic enhancement of local electric fields near metal nanoparticles. Strong spatial localization of electron-oscillations at the plasmon resonance frequency leads to a huge increase in the local electric field. Plasmonic enhancement near metal nanomaterials is explained by electromagnetic theory and is confirmed by FDTD calculations. We expect that the potential use of plasmonics will be explored further over time, especially the use of metal nanoparticles.

Despite many significant achievements in the synthesis and application of plasmonic metal nanoparticles, there are still many challenging issues in synthesis methods and optical sensing applications of metal nanoparticles.

- Compared with semiconductor nanoparticles, plasmonic metal nanoparticles have advantages and limitations [212]. The advantages of metal nanoparticles are their high sensitivity, simple fabrication process, tunable shape, and size. The limitations include high cost, low stability, and poor biocompatibility.
- Plasmonic metal nanoparticles can be used in theranostics for cancer diagnostics and treatment. Various gold-based organic and inorganic nanoparticles have attracted increasing research attention due to the feasibility of surface functionalization, excellent tumor specificity, high drug-loading capacity, and biocompatibility [108,213]. Platinum and rhodium nanoparticles are promising for applications in deep-UV SERS, therapeutics, diagnostics, and biosensing fields [214,215].
- Plasmonic metal nanoparticles can be used for single-molecule and single-cell detection purposes [105,216]. They can be extremely useful in the sensing of pathogens [121,217] and intracellular components [218]. Metal nanoparticle-based SERS sensing is widely used in environmental contaminant monitoring [123]. Another challenging issue is metallic nanoparticle application as nanocarriers for drug delivery systems [219].
- This paper highlights the promising potential of optical sensors over a wide range of tasks. It provides a detailed analysis for optical sensing applications of plasmonic metal nanoparticles, and reveals the prospects for optical sensing applications of metal nanoparticles in the UV and visible ranges. In the near future, optical sensing applications of metal nanoparticles by IR- and far-IR-plasmonics will be reviewed.

## Figures and Tables

**Table 1:** Representative results for synthesis methods and optical sensing applications of the rhodium- and platinum-based nanomaterials.

	Synthesis Methods	Optical Sensing Applications	Ref.
**Rh-based nanomaterials**	• Chemical reduction of RhCl${}_{3}$ with alkaline 2,7-DHN on DNA scaffolds under UV-light irradiation.	• SERS sensing by ultra-small, self-assembled RhNPs on DNA scaffold.	[78]
	• Co-reduction of the metal precursors by polyol at elevated temperature.	• SERS sensing by Ag–Rh nanomaterials.	[81]
	• Chemical reduction of RhCl${}_{3}$ with NaBH${}_{4}$ on DNA scaffolds.	• SERS sensing by Rh@DNA NPs.	[82]
	• Polyol synthesis. (Chemical reduction of Na${}_{3}$RhCl${}_{6}$ by ethylene glycol).	• SERS sensing by RhNP multipods (Tripod and tetrapod RhNPs).	[79]
	• Electron-beam physical-vapor-deposition.	• MEF sensing by RhNP substrates.	[84]
**Pt-based nanomaterials**	• Chemical reduction of H${}_{2}$PtCl${}_{6}$ and physical ablation from bulk Pt.	• SERS sensing by PtNPs.	[90]
	• Electrodeposition of Pd or Pt through a template of self-assembled polystyrene latex spheres onto a suitable conducting surface.	• SERS sensing by structured Pt and Pd surfaces.	[95]
	• Chemical reduction of H${}_{2}$PtCl${}_{6}$ with sodium citrate, sodium borohydride, and L-ascorbic acid.	• Gap-enhanced Raman scattering of 4-ABT positioned in the gaps formed by a flat Ag substrate and 20–150 nm PtNPs.	[91]
	• Synthesis of Pt nanocubes, Pt nanospheres, Au core Pt shell (Au@Pt), and Au core Pd shell (Au@Pd) nanoparticles from chemical reduction.	• Shaping and shelling PtNPs and PdNPs for UV–SERS.	[94]
	• Chemical reduction of H${}_{2}$PtCl${}_{6}$ using a multistep seed-mediated approach.	• SERS sensing of melamine by PtNPs with different shapes and sizes.	[96]
	• Deposition on the n-type Si substrate by galvanic displacement method.	• SERS sensing by Pt- and Pd-nanostructures.	[80]
	• Core-shell Au@Pt NPs by seed-mediated chemical method. (Synthesis of Au seeds by the Frens method, and then deposition of the Pt shells on the Au surface by in situ reduction. )	• Simultaneous identification of multiple mitochondrial ROS in living cells by a SERS-based nanoprobe (core-shell Au@Pt NPs).	[101]
	• Synthesis of AuNPs by citrate reduction of HAuCl${}_{4}$, and then synthesis of core-shell Au@Pt NPs by reduction of H${}_{2}$PtCl${}_{6}$ with ascorbic acid.	• Ag@Pt NPs as an enzymatic reporter to identify microcystin-leucine arginine antibodies.	[99]
	• Chemical reduction of PtCl${}_{2}$ in ethylene glycol under boiling for 3 h by Lewera polylene method.	• SERS and TERS studies for the selective adsorption of PBA–PA derivatives on the surface of PtNPs.	[100]

**Table 2:** Representative results for synthesis methods and optical sensing applications of the gold- and silver-based nanomaterials.

	Synthesis Methods	OpticalSensingApplications	Ref.
**Gold-based nanomaterials**	• Citrate reduction of gold precursor; Deposition of AuNPs on the ordered tellurium nanowire template.	• Efficient SERS platform by highly ordered gold nanowire arrays. ➀ Anisotropic NP arrays. ➁ Polarization-dependent SERS.	[114]
	• Oblique incidence physical-vapor-deposition on pre-patterned rippled substrate by low energy ion irradiation.	• Anisotropic SERS of gold nanowire arrays and NP chains. ➀ Comparative study of nanowire arrays and NP chains. ➁ Polarization-dependent SERS.	[103]
	• Chemical reduction of gold precursor with NaBH${}_{4}$.	• Polarization-dependent SERS in gold nanoparticle-nanowire system.	[115]
	• Vapor transport method.	• SERS sensing of benzenethiol (BT) by a single gold nanowire on a gold film. ➀ Nano-gap-enhanced Raman scattering. ➁ Polarization-dependent SERS.	[102]
	• Citrate reduction method.	• Single molecule and single living cell detections by AuNP-based SERS.	[106]
	• Citrate-stabilized AuNPs.	• In vivo tumor targeting and SERS detection by scFv, EGFR-conjugated AuNPs.	[108]
	• CTAB-stabilized Au nanocubes by NaBH${}_{4}$ reduction of gold precursor.	• SERS sensing of human immunodeficiency virus (HIV) by Au nanocubes.	[116]
	• Citrate reduction method.	• SERS sensing of food-borne pathogens by biorecognition element-conjugated AuNPs.	[117]
	• Numerical simulation study.	• Influence of size, shape, and dielectric environment on the optical properties of metal NPs.	[23]
**Silver-based nanomaterials**	• Chemical reduction of silver precursor with ascorbic acid.	• Efficient SERS platform by self-assembled AgNP monolayer.	[118]
	• Plasma reduction of silver precursor.	• SERS sensing by AgNPs loaded on a polyester fabric by plasma jet printing.	[119]
	• Citrate reduction of silver precursor; Deposition of AgNPs on graphene nanosheets.	• SERS sensing of the freshness of fruits and vegetables by AgNPs and AuNPs supported on graphene nanosheets.	[113]
	• Citrate reduction method.	• Cancer detection by AgNP-based SERS.	[120]
	• Citrate reduction method.	• Single-molecule detection of rhodamine 6G by AgNP-based SERS.	[105]
	• Fabrication of Ag nanowires in highly ordered porous aluminum oxide (PAO) template by AC electrodeposition.	• Efficient SERS platform by Ag nanowire bundles.	[111]
	• Review for synthesis methods and plasmonic sensing applications.	• Synthesis methods and SERS/SEF sensing of AgNPs.	[109]

**Table 3:** Synthesis methods of plasmonic metal nanoparticles.

SynthesisMethods	SpecificSynthesisMethods
Physicalsynthesis	• Sputtering
	• Electron beam evaporation
	• Thermal evaporation
	• Radiolysis
	• Plasma synthesis
	• Laser ablation
	• Ultrasonication
	• UV photolysis
	• Laser pyrolysis
	• Spray pyrolysis
	• Mechanical milling
	• Lithography
Chemicalsynthesis	• Chemical reduction of metal salts
	• Polyol synthesis
	• Sol-gel synthesis
	• Vapor transport method
	• Chemical vapor deposition
	• Plasma-enhanced chemical vapor deposition
	• Electrochemical synthesis
	• Phytochemical synthesis
	• Microemulsion synthesis
Greensynthesis	• Plant extract-assisted synthesis
	– Leaves, Flowers, Fruits, Roots
	• Microorganism-assisted synthesis
	– Fungi, Yeast, Bacteria, Viruses, Actinomycetes
	• Algae-based synthesis

## Data Availability

Not applicable.
